# Proteomic analysis of extremely severe hand, foot and mouth disease infected by enterovirus 71

**DOI:** 10.1186/1471-2334-13-383

**Published:** 2013-08-20

**Authors:** Li Deng, Hong-Ling Jia, Chao-Wu Liu, Yu-Fen Xu, Li-Jia Mao, Chun-Hui He, Gen-Quan Yin, Jun-Hong Lin, Jian-Ping Tao, Li Zhu

**Affiliations:** 1 Guangzhou Women and Children′s medical center, Guangzhou 510120, Guangdong, China; 2 Key Laboratory of Functional Protein Research of Guangdong Higher Education Institutes, Institute of Life and Health Engineering, College of Life Science and Technology, Jinan University, Guangzhou, Guangdong, 510632, China; 3 School of Public Health and Tropical Medicine, Southern Medical University, Guangzhou 510515, Guangdong, China; 4 Guangdong Institute of Microbiology/Guangdong Provincial Key Laboratory of Microbial Culture Collection and Application, Guangzhou 510070, Guangdong, China

**Keywords:** Extremely severe HFMD, MALDI-TOF/TOF MS, Proteomic analysis

## Abstract

**Background:**

To clarify the molecular mechanisms that participate in the severe hand, foot and mouth disease (HFMD) infected by Enterovirus 71 and to detect any related protein biomarkers, we performed proteomic analysis of protein extracts from 5 extremely severe HFMD children and 5 healthy children.

**Methods:**

The protein profiles of them were compared using two-dimensional electrophoresis. Differentially expressed proteins were identified using mass spectrometry. Functional classifications of these proteins were based on the PANTHER. The interaction network of the differentially expressed protein was generated with Pathway Studio.

**Results:**

A total of 38 differentially expressed proteins were identified. Functional classifications of these proteins indicated a series of altered cellular processes as a consequence of the severe HFMD. These results provided not only new insights into the pathogenesis of severe HFMD, but also implications of potential therapeutic designs.

**Conclusions:**

Our results suggested the possible pathways that could be the potential targets for novel therapy: viral protection, complement system and peroxide elimination.

## Background

Human enterovirus 71 (EV71) and coxsackievirus A16 (CA16) are two predominant pathogens causing HFMD. EV71 is more associated with major outbreaks and causes complications of greater severity, severe neurologic symptoms, and higher rates of mortality than other enteroviruses [1-4]. Outbreaks of EV71 have been reported since 1969 [1]. Especially since the late 1990s, a significant increase in EV71 epidemics is observed, creating a serious threat to public health throughout the Asia-Pacific region [5-7]. For example, in 2007, more than 80,000 HFMD cases were reported with dozens of deaths in mainland China [8,9].

The complications of severe HFMD include pneumonia, myocarditis, encephalitis, brain-stem encephalitis and acute flaccid paralysis. High mortality and severe sequelae can be anticipated when the disease is complicated by neurogenic pulmonary edema, rapid disease progression [10,11].

Only a small fraction of the mild cases further develop to the severe and fatal cases. However, these cases often progress rapidly and cause failure and deaths before effective treatment take place. As the rapid progression of symptoms in fatal cases, rapid diagnosis is essential for severely EV71-infected patients to provide timely treatment [12]. EV71 virus can be detected by testing viral antigens, viral genomic RNA and antiviral antibodies in HFMD patients [13-15], however, no quick method has been established to distinguish mild HFMD from severe HFMD during the rapid progression of symptoms in fatal cases of EV71 infection.

Two-dimensional gel electrophoresis (2-DE) and MALDI-TOF/TOF mass spectrometry have been extensively applied to identify differentially expressed proteins between different stages of disease, as well as those in healthy individuals, in a wide variety of biological systems [16]. This strategy is able to detect potential biomarkers for disease diagnosis, progression and therapy while does not require much knowledge background of the disease. In this study, we used comparative proteomics to systemically study the protein profile changes in serum of extremely severe HFMD. The proteome map presented here may serve as an important basis for future studies.

## Methods

### Preparation of serum samples

Blood samples from 5 HFMD children (five extremely severe) according to the ‘foot and mouth disease prevention control guide’ (2008 Edition) issued by the Ministry of Health, China (http://www.moh.gov.cn/publicfiles/business/htmlfiles/mohbgt/s9511/200805/34775.htm), were randomly selected to be analyzed with 2-D Gel Electrophoresis and MALDI-TOF-MS and clinical symptoms and laboratory test (EV71 nucleic acid detection kit) confirmed that the EV71 infections caused all these HFMD cases. In addition to meeting the above symptoms, these children all had encephalitis, pulmonary hemorrhage, and mechanical ventilation and other clinical symptoms. They were proven to have no other disease after a systematic check in the hospital. Another five blood samples from healthy children were collected as controls. The protocols applied in this study were approved by the Ethical Committee of Guangzhou Women and Children′s medical center. The parents of all participants of this study provided written informed consent. A pooled sample consists of equal amounts of each of 5 experimental samples. Blood sample was separated by centrifugation at 1000 g for 20 min. Aliquots of serum were collected and stored at -80°C until ready for use. Serum samples were processed using the ProteoExtract^®^ Albumin/IgG Removal Kit (Merck, New jersey, USA) that selectively removes IgG and albumin from the serum sample. Serum samples were handled according to the manufacture’s instructions. To purify the protein extraction and determine the final protein concentration, the 2-D Clean-up Kit (GE healthcare, UK) and Bradford protein assay kit (Bio-Rad, USA) were used sequentially, following the manufacturer’s instructions.

### Two-dimensional (2D) gel electrophoresis

Total proteins were mixed up to 250 μL of rehydration solution (8 M urea, 20 mM DTT, 2% CHAPS, and 0.5% IPG buffer). For each sample 300 μg of total protein were used for the experiment. 2-DE gel was performed with Amersham Biosciences IPGphor IEF System and Hoefer SE 600 (GE Healthcare) electrophoresis units using a 13-cm immobilized pH 3–10 nonlinear gradient IPG strips according to the protocol suggested by the manufacturer. The rehydration step was performed for 10 h at low voltage of 30 V. IEF was run by following a step-wise voltage increase protocol: 500 and 1000 V for 1 h each and 5000–8000 V for about 10 h with a total of 64 kVh. After IEF, the strips were subjected to two-step equilibration in equilibration buffers (6M urea, 2% SDS, 30% glycerol and 50 mM Tris–HCl pH 6.8) with 1% DTT w/v for the first step, and 2.5% iodoacetamide (w/v) for the second step. The equilibrated gel strip was placed on top of a 12.5% SDS-PAGE gel, sealed with 0.5% agarose containing a little bromophenol blue. SDS-PAGE was carried out at a constant current of 15 mA for 30 min per gel and then 30 mA per gel until the bromophenol blue reached the bottom of the gels. Proteins were detected by a silver nitrate staining.

### Image analysis

Analytical gels were scanned on an Image Scanner (GE healthcare) at 300 dpi with 12-bit gray scale levels in tagged image file format (TIFF), images were analyzed using the ImageMaster 2D Platinum (GE Healthcare). All gels in the analyses were scanned with identical parameters. The individual spots of each gel were detected by their boundaries and the spot volumes corresponding to the protein abundance were calculated automatically. Each spot intensity volume was processed by background subtraction and total spot volume normalization. The resulting spot volume percentage was used for comparison. Only those spots that have statistical significance in differential expression, as judged by software analysis of the silver-stained gels, were excised from gels for analysis by MS.

### In-gel digestion

Each gel piece was rinsed three with deionized water, destained in a 1:1 solution of 30 mM potassium ferricyanide and 100 mM sodium thiosulfate and then equilibrated in 50 mM ammonium bicarbonate to pH 8.0. After hydrating with acetonitrile and drying in a Speed Vac. The gel spots were rehydrated in a minimal volume of trypsin (Promega, USA) solution (20 μg/ml in 25 mM NH_4_HCO_3_) and incubated at 37°C overnight. The supernatants were transferred into a 200 μL microcentrifuge tube and the gels were extracted once with extraction buffer (67% acetonitrile containing 2.5% trifluoroacetic acid). The peptide extract and the supernatant of the gel spot were combined and then completely dried in a Speed Vac centrifuge.

### Protein identification and data analysis

After digestion, tryptic peptides were lyophilized and resuspended in 2 μL of a 30% acetonitrile/0.1% TFA solution. 0.8 μL of the samples were spotted onto the MALDI sample target plate, followed by 0.4 μL of a saturated matrix solution of a-cyano-4-hydroxycinnamic acid prepared in 50% acetonitrile/0.1% TFA.

Peptide mass spectra were obtained on a MALDI-TOF/TOF mass spectrometer (4800 Proteomics Analyzer, Applied Biosystems, Foster City, CA, USA) in the positive ion reflectron mode. After an external calibration with a mixture of Gradykinin (Mr, 904.458), angiotensin I (Mr, 1296.685), Glul-Fibrinopeptide (Mr, 1570.677), ACTH clip 1–17 (Mr, 2093.08), ACTH clip 18–39 (Mr, 2465.199), ACTH clip 7-38(Mr, 3657.929), spectra were obtained in the mass range between 900 and 3500 Da with 500 laser shots. For each sample spot, a data dependent acquisition method was created to select the seven most intense peaks(S/N>50), excluding those from the matrix, due to trypsin autolysis or acrylamide peaks, for subsequent MS/MS data acquisition. MS/MS spectra were acquired with 1200 laser shots in the mass range from 10 Da to the mass of parent ion using an interpretation method present on instrument software, where the seven most intense peaks were selected and MS/MS spectra were generated automatically.

Database search spectra were processed and analyzed by the Global Protein Server Workstation (Applied Biosystems, Foster City, CA, USA), which uses internal Mascot (Matrix Science Ltd, UK)software for searching the peptide mass fingerprints and MS/MS data. Searches were performed against the uniport database with the taxonomy of Homo sapiens. Mass searches were performed using mass tolerance settings of 100 ppm for the precursor and 0.2 Da for the fragment masses. The following parameters were used in database searching: fixed modification, carbamidomethyl (cysteine), variable modification, oxidation (methionine), Up to one missed trypsin cleavage was allowed. To ensure a reliable identification, the results from both the MS and MS/MS spectra were used in the database search. Protein identification was accepted when the score reported by the Mascot search routine was higher than 62 and, whenever possible, confirmed with MW/pI values.

### Protein categorization and network construction

Identified proteins were classified based on the PANTHER (Protein ANalysis THrough Evolutionary Relationships) system (http://www.pantherdb.org), a unique resource that classifies genes and proteins by their functions. The PANTHER ontology, a highly controlled vocabulary (ontology terms) by molecular function, protein class and biological process was used to categorize proteins into families and subfamilies with shared functions.

The interaction network of the differentially expressed protein was generated with Pathway Studio version 5.0 software (Ariadne Genomis, Rockville, MD) and Resnet 5 database. Common upstream regulators or downstream targets of multiple proteins were identified by using this software that facilitated the process of selecting potential mechanisms and key factors from the large number of differentially regulated proteins.

### Western blot analysis

Protein extracts from serum of normal and extremely severe HFMD were separated by SDS-PAGE (12–15% acrylamide) and then electroblotted onto PVDF membranes. The membranes were incubated with Keratin type II cytoskeletal 6C(KRT6C),Serum amyloid P-component(APCS), Apolipoprotein A-1(APOA1) and Peroxiredoxin-2(PRDX2) antibodies at 4°C overnight, followed by incubation with corresponding secondary antibodies at room temperature for 2 h. Specific protein bands were visualized with the SuperSignal chemiluminescence system (ECL, Pierce, USA) and imaged by autoradiography.

## Results

### Comparative proteomic analysis of extremely severe HFMD

**Figure 1:**
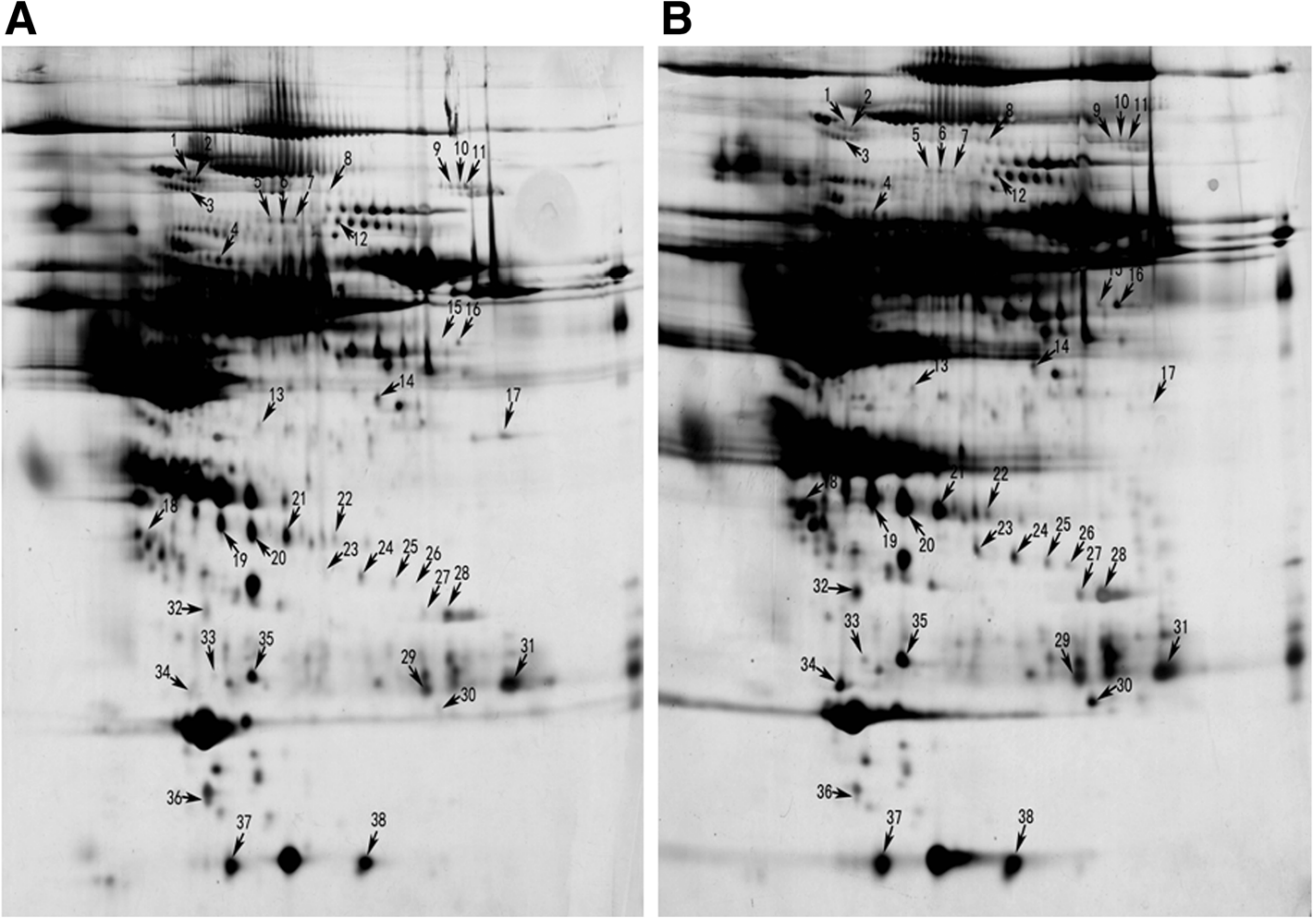
**2-DE analysis of differentially expressed protein spots between healthy children (A) and extremely severe HFMD patients (B).** The gels were visualized by silver staining. Differentially expressed proteins are marked using numbers, which correspond to the Table 1.

Same amount of serum proteins of normal persons and extremely severe HFMD patients were depleted of the high-abundance proteins like albumin and IgG to reduce their disturbance to the detection of low-abundance proteins. The samples were then subjected to 2-DE followed by silver staining (Figure 1A for controls and Figure 1B for patients). The gels were scanned and compared with ImageMaster 2D Platinum software to identify the protein spot variations (Table 1). We identified 38 differentially expressed proteins from the 2-D gels and identified them by MALDI TOF/TOF MS. Among these, 24 proteins were up-regulated and 14 proteins were down-regulated in the extremely severe HFMD samples compared with the level in control samples. Two proteins appeared exclusively in the extremely severe HFMD patients, not in the healthy children.

**Table 1:** **Identification of differentially expressed proteins of extremely severe HFMD patients by MALDI**-**TOF**/**TOF**

Spot no*	**Protein name**	**Accession NO.**	**Protein MW(kDa)**	**Protein pI**	**Protein score C.1.%**	**Ratio (S/N)**
1	Ceruloplasmin	P00450	122.98	5.44	99.96	-2.31
2	Ceruloplasmin	E9PFZ2	109.49	5.49	100	-3.006
3	35 kDa inter-alpha-trypsin inhibitor heavy chain H4	F5H194	98.55	6.21	100	-3.574
4	Inter-alpha-trypsin inhibitor heavy chain H2	P19823	106.85	6.4	100	-1.889
5	Serum albumin	E7ESU5	72.46	5.82	100	-3.598
6	Serum albumin (Fragment)	H0YA55	53.08	6.45	100	-5.505
7	Serum albumin (Fragment)	H0YA55	53.08	6.45	100	-3.019
8	C2ORF3 variant 2	A4UHQ9	28.03	5.08	96.214	1.887
9	Plasminogen	P00747	93.25	7.04	99.995	-2.122
10	Plasminogen	P00747	93.25	7.04	100	-2.81
11	Plasminogen	P00747	93.25	7.04	99.987	-2.981
12	Alpha-2-macroglobulin	P01023	164.61	6.03	100	-1.5799
13	Pigment epithelium-derived factor	P36955	46.45	5.97	96.992	1.621
14	Hemopexin	P02790	52.38	6.55	100	-1.566
15	Ig alpha-2 chain C region	P01877	37.30	5.71	100	1000000
16	Cdna FLJ55673 highly similar to complement fator B	B4E1Z4	143.19	6.82	99.862	5.1141
17	Complement C3	P01024	188.57	6.02	99.63	-7.118
18	Clusterin alpha chain (Fragment)	H0YAS8	16.22	5.51	100	5.212
19	Haptoglobin beta chain	I3L0D3	31.67	8.48	100	1.612
20	Haptoglobin beta chain	I3L0D3	31.67	8.48	100	1.845
21	Haptoglobin beta chain	I3L0D3	31.67	8.48	100	1.8315
22	MTHFSD Uncharacterized	B7ZLC2	24.26	6.97	95.54	2.6638
23	Haptoglobin beta chain	I3L0D3	31.67	8.48	100	3.569
24	Isoform 1 of Ficolin-3	O75636	33.40	6.20	100	1.8743
25	Isoform 2 of Ficolin-3	O75636	32.11	6.36	100	1.9465
26	Apolipoprotein L1	E9PF24	42.19	5.58	96.777	1000000
27	complement component 4B preproprotein	B4E344	194.17	6.89	99.987	2.1058
28	DADB-112B14.11 Complement component 4B	B0UZ85	194.17	6.89	100	2.404
29	IGK@ protein	Q6P5S8	26.04	5.94	99.398	2.004
30	Peroxiredoxin-2	P32119	22.05	5.66	100	9.686
31	Ig kappa chain V-III region WOL	P01623	11.85	9.07	100	2.489
32	Keratin, type II cytoskeletal 1	P04264	66.17	8.15	99.112	2.4587
33	Keratin, type II cytoskeletal 6C	P48668	60.27	8.09	100	2.5654
34	Apolipoprotein A-1	P02647	30.76	5.56	100	4.3662
35	Serum amyloid P-component	P02743	25.49	6.10	100	3.863
36	Retinol-binding protein 4	P02753	23.34	5.76	100	-2.876
37	Haptoglobin beta chain	H0Y300	22.53	5.96	97.871	1.527
38	HPR 47 kDa protein	P00738	47.38	6.28	100	1.7345

“1000000”—only appeared in the extremely severe HFMD patients, but not appeared in the healthy children.

*Spot numbers correspond to the numbers marked in Figure 1.

Note: some proteins showed in the 2-DE as multiple spots, probably due to their isoforms and/or modifications.

### Validation of differentially expressed proteins in extremely severe HFMD

**Figure 2:**
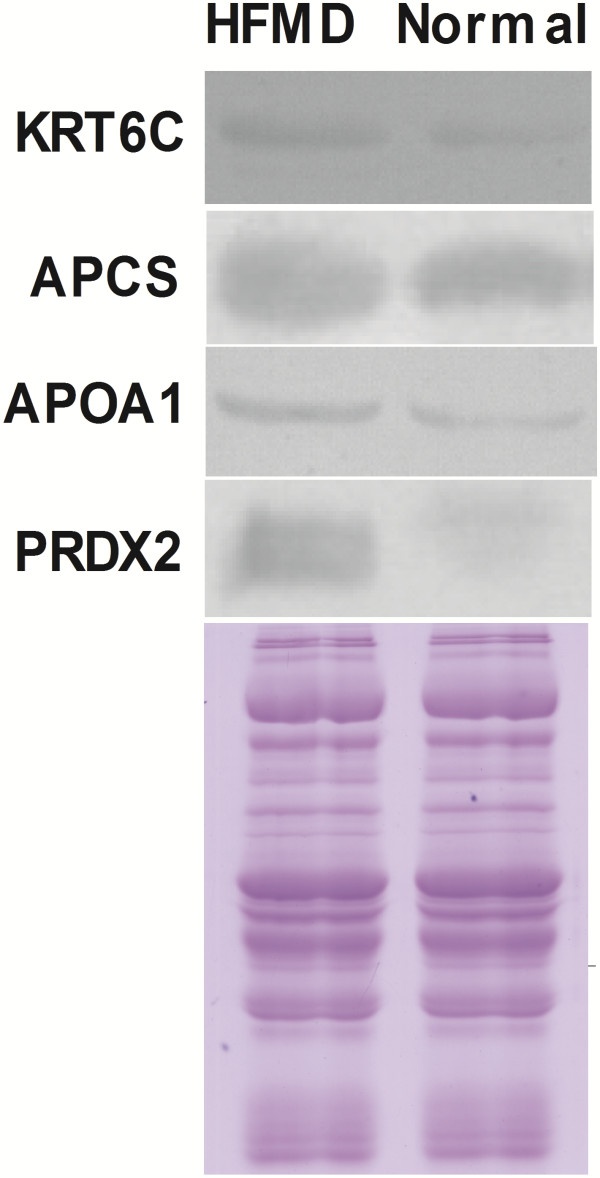
**Western blot validation of four proteins (KRT6C, APCS, APOA1 and PRDX2) in extremely severe HFMD serum samples, and the Coomassie -stained blot (below) is the loading control.**

To verify the differential expression of proteins in extremely severe HFMD, we used western blot analysis to examine the expression of three proteins, including KRT6C, APCS, APOA1 and PRDX2. The Western blotting results showed the same pattern of expression as obtained from 2-DE analysis. That is, they are significantly increased in extremely severe HFMD (Figure 2).

### Functional categories and biological interaction networking of identified proteins

**Figure 3:**
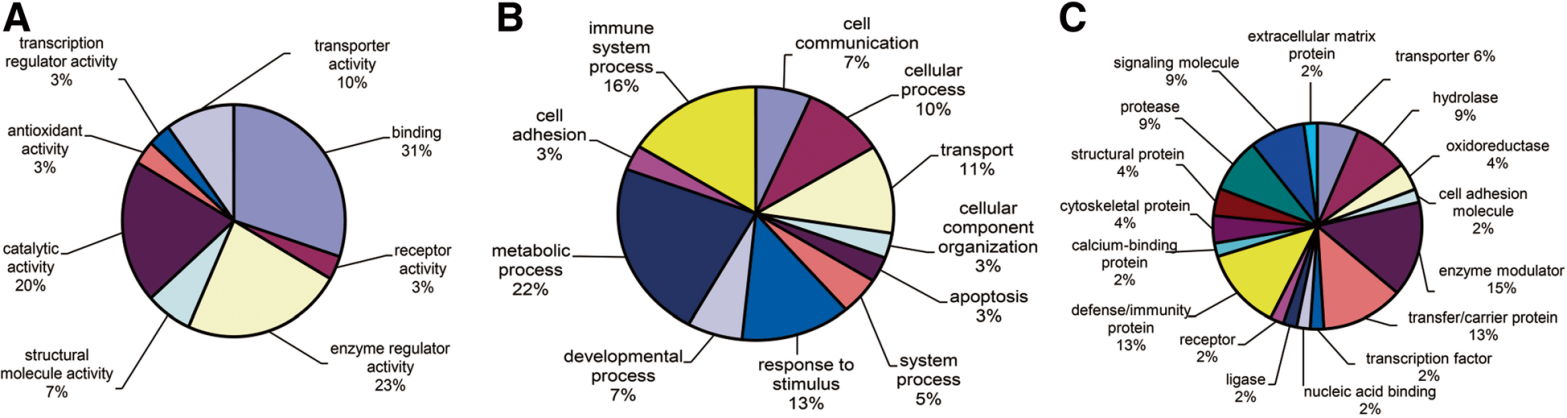
Functional classification of identified differentially expressed proteins from extremely severe HFMD serum samples according to their (A) molecular functions, (B) biological processes and (C) protein class.

In order to understand the biological relevance of the changes in protein expression in extremely severe HFMD, PANTHER [17] software was used to categorize the identified proteins according to their molecular functions, biological process and protein class (Figure 3). The result showed an enrichment of the binding proteins (31%) and enzyme regulators (23%) (Figure 3A). The patients had altered metabolic process (22%) and enhanced response to stimulus (13%), indicating that the body was in a stress condition (Figure 3B). The immune system was also stimulated to counteract the lesion caused by the virus (16%) (Figure 3B). The enrichment is not protein-class specific, indicating a complex response in the case of HFMD (Figure 3C).

**Figure 4:**
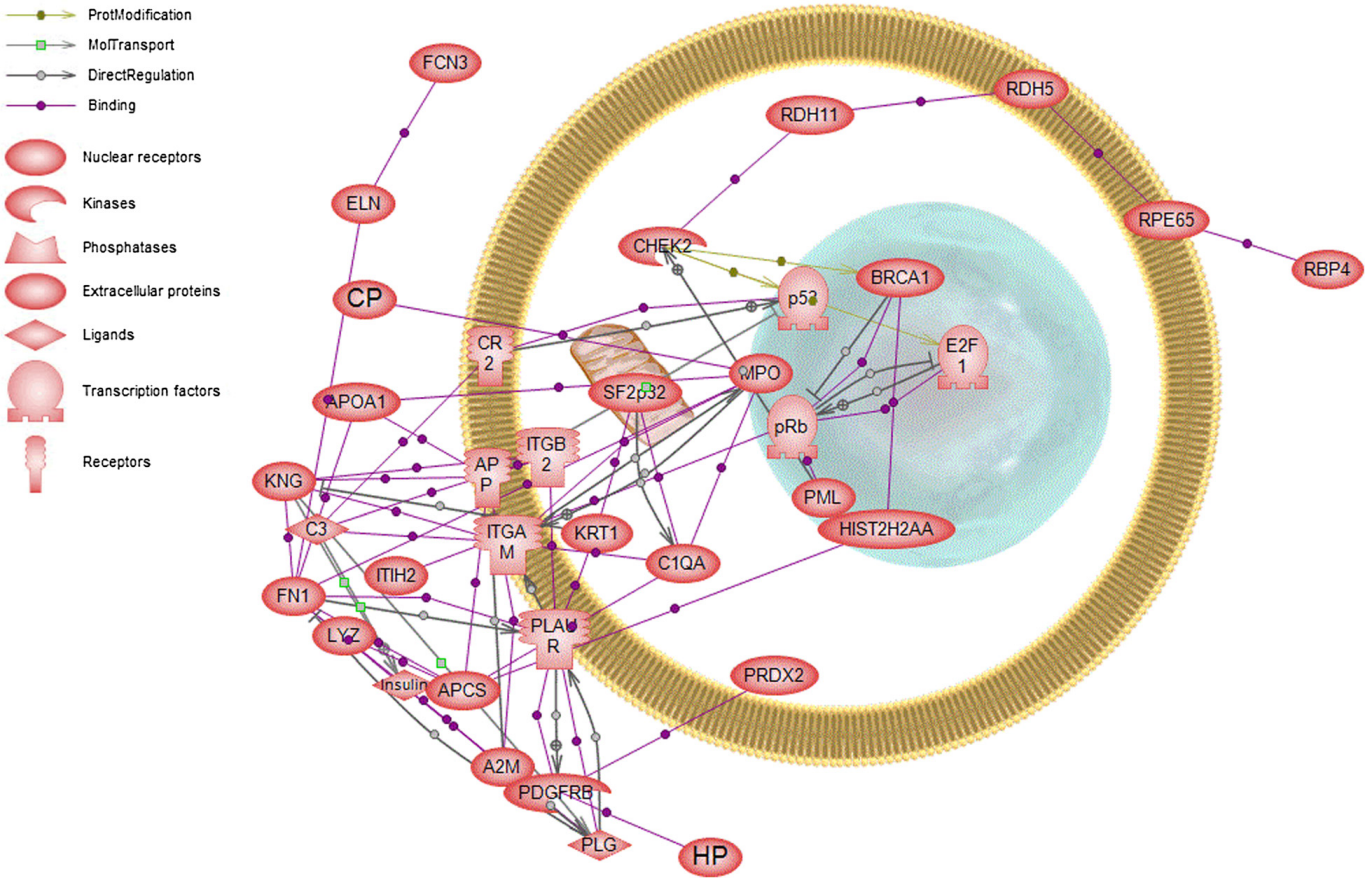
**Pathway Studio analysis of the differentially expressed proteins of extremely severe HFMD serum samples.** Proteins identified in Table 1 were imported into PathwayAssist and an interaction map was created. Each node represents either a protein entity or a control mechanism of the interaction. Shown are proteins that either bound directly to another identified protein or to another identified protein via one other protein. The legend of the interaction network is summarized on the right of the figure.

Bioinformatic analysis using Pathway Studio [18] software enabled the characterization of biological association networks related to these differentially expressed proteins (Figure 4). The proteins that could be networked were linked by various relationships such as expression regulations, modifications and protein interactions. The network showed complex interactions of extracellular and intracellular proteins, as well as nuclear proteins and a mitochondria protein, indicating a complex signaling network involving transcription regulation and mitochondria signaling pathways.

## Discussion

There are no effective antiviral drugs and vaccines available against HFMD, therefore the timely and accurate diagnosis and monitoring is crucial for the treatment. In the present study, we took advantage of proteomics to identify potential markers which reflect the status of HFMD. The goals were to (i) define a set of differentially expressed proteins that may be used as potential biomarkers to facilitate the severe HFMD diagnosis and monitoring of therapy effectiveness and (ii) gain a deeper understanding of the early pathological processes in severe HFMD.

We identified 38 differentially expressed spots which were detected in the serum of extremely severe HFMD patients compared with the healthy controls (Table 1). The known functions of these proteins are summarized in Figure 2. These proteins have previously been identified using proteomics approaches in mild and severe HFMD children [19]. However, a change in their expression in relation to extremely severe HFMD had never been previously reported. Extremely severe HFMD represent the most acute and serious situation of this disease and usually life-threatening. Biomarkers for this stage can serve as a warning signal for an urgent treatment. A variety of cellular functions was covered from the identified proteins, including enzyme modulator, infection defense/immunity, signaling molecule, cytoskeleton maintenance, metabolism etc., reflecting the human body facing acute lesions.

Our study showed an apparent down regulation of Complement C3(C3), inter-alpha (globulin) inhibitor H2 (ITIH2), serum albumin (ALB), plasminogen (PLG) and retinol binding protein 4 (RBP4) [20-22]. The function of these proteins typically involve in virus invasion protection and elimination; histamine release and muscle smooth; colloidal osmotic pressure of blood regulation and retinol delivery. Thus, down regulation of the proteins indicated that the viral infection suppressed these reactions from HFMD patients, facilitating the disease progression. Inhibition of the viral protection enhances the virulence of the virus, leading to extremely severe symptoms and even fatal lesions. This suggested that antiviral therapies may help to control the disease progression in the extremely severe phase.

Previous studies have noted that many diseases, including the neurological diseases known as transmissible spongiform encephalopathies (TSEs), are due to inflammatory injury [23]. EV71 infection can induce complement activation and an inflammatory response of the central nervous system. Inappropriate or excessive activation of the complement system can result in severe inflammation or tissue injury [24,25]. It was known that complement C3 plays a key role in the activation of the complement system [26]. From our study we found that the C3 was the most down regulated protein of the extremely severe HFMD. These information together with our results imply the activation of complement system due to EV71 infection and inflammatory is associated with complement activation. Whilst antiviral therapy is one possible approach, it is perhaps more important to inhibit the inflammatory response. Complement inhibition should greatly reduce the inflammatory reaction or tissue injury resulting from excess complement activation.

Peroxiredoxin-2 (PRDX2) was found extremely significant increased in extremely severe HFMD patient. It plays an important role in eliminating peroxides generated during metabolism. It participates the signaling cascades of growth factors and tumor necrosis factor-alpha by regulating the intracellular concentrations of H_2_O_2_. It is one of the most important antioxidant enzymes in humans [27,28]. Previous studies showed that antioxidant enzymes activity, increased in severe HFMD patients and decreased significantly after vincristine–adriamycin– dexamethasone therapy [29]. Therefore, we speculate that upregulation of PRDX2 may play significant roles in pathogenesis of severe HFMD and could be used as the potential drug targets.

## Conclusions

We reported, for the first time, the analysis in a proteome level to identify proteins that change in expression in extremely severe HFMD. These proteins have the potential to be used, probably in combination, as potential molecular markers to monitor the progression of HFMD treatment. The results also suggested the crucial pathways in the development of the disease in extremely severe phase, providing important hints for the functional studies and therapy design of HFMD. Further validation with a much larger sample size is necessary to determine the biomarker set for clinical applications.

## Competing interests

The authors declare that they have no competing interests.

## Authors’ contributions

LD, HLJ and CWL created the concept and design of this study. CHH, GQY, JHL and JPT participated in sample diagnosis and collection. YFX and LJM performed the experiments. HLJ and LZ were responsible for the statistical analysis. LD, HLJ and LZ drafted, revised and edited the manuscript. All authors read and approved the final manuscript.

## Pre-publication history

The pre-publication history for this paper can be accessed here:

http://www.biomedcentral.com/1471-2334/13/383/prepub
